# How should the results of Alzheimer's disease treatment trials be communicated to colleagues, patients, and families?

**DOI:** 10.1002/trc2.70330

**Published:** 2026-10-13

**Authors:** Jemma Hazan, Simone Salemme, Kathy Y. Liu, Robert Howard

**Affiliations:** ^1^ Division of Psychiatry University College London London UK; ^2^ North London NHS Foundation Trust London UK; ^3^ Department of Biomedical, Metabolic and Neural Sciences University of Modena and Reggio Emilia Modena Italy; ^4^ International School of Advanced Studies University of Camerino Camerino Italy

**Keywords:** Alzheimer's disease, clinical trial, dementia, disease modification, time savings

## Abstract

The approval of amyloid‐lowering therapies for Alzheimer's disease (AD) has increased the importance of clearly and precisely communicating complex clinical trial findings to patients, caregivers, clinicians, and the wider public. Because the clinical relevance of the modest treatment effects observed on cognitive and functional rating scales, which served as primary trial outcome measures, is uncertain and difficult to interpret, these effects have been translated into alternative quantitative interpretations, such as relative slowing of decline and time‐saved estimates, and supplemented with qualitative descriptions and patient or caregiver testimonials. In this perspective, we examine the advantages and limitations of these approaches, with a particular focus on time‐saved approaches, and discuss the assumptions required to move beyond reliance on directly observed primary outcome data from clinical trials. We argue that alternative methods of communicating treatment effects may improve accessibility but introduce additional uncertainty and may lead patients and clinicians to overestimate the size of benefit because they will not appreciate some of the assumptions on which these methods are based. Clear and accurate communication of treatment benefits, risks, limitations, and evidential uncertainty is essential to support informed decision making and maintain public trust in AD research and treatment.

## Highlights

- Clinical trial results are frequently translated beyond their observed outcomes.
- Time‐saved estimates and relative slowing require careful interpretation.
- Communication should distinguish measured, modeled, and inferred treatment effects.

## 1 INTRODUCTION

Clear and precise communication of the results of Alzheimer's disease (AD) clinical trials to the scientific community and to the wider public is a fundamental responsibility, as these results inform decision making by affected patients and their caregivers, clinicians, policy makers, and other stakeholders. AD is one of the most feared health conditions, responsible for ≈ 32 million cases of dementia worldwide and imposing a substantial public health burden.^1^, ^2^ The development of a cure remains the top research priority for the public^1^ and considerable resources have been devoted to identifying pharmacological agents that may alter, delay, or prevent disease progression.

Between November 2022 and July 2023, two phase 3 clinical trials of amyloid‐lowering therapies reported positive results, leading to the marketing approval of donanemab and lecanemab internationally.^3^, ^4^ This garnered unprecedented public attention and saw the translation of clinical trial data, designed primarily to inform regulators’ licensing decisions, into descriptions that would make potential treatment benefits more easily understood and imbued them with greater clinical relevance than reported small differences on main outcome measures might suggest. Such translations have the potential to generate misleading interpretations that have not always been subject to sufficient scrutiny.

The way in which trial results are communicated will shape patient and family expectations, influence treatment decisions, and guide policy and service planning. It is therefore important to distinguish between statements about efficacy that are directly supported by the observed trial evidence, reasonable interpretations of that evidence, claims that rely on additional assumptions, and claims that are not supported by data.

In this article, we discuss the approaches used to interpret and communicate the primary outcomes of recent AD clinical trials to clinician and lay audiences, examine the complexities and potential pitfalls involved in translating clinical trial data for non‐specialist audiences, and propose that claims should be classified according to their evidential distance from the trial data: directly observed results, derived quantitative transformations, model‐dependent estimates, patient‐salient extrapolations, and unsupported or incorrect claims.

## 2 THE CHALLENGES IN INTERPRETING TRIAL OUTCOMES

The use of numerical information has been shown to improve patient and clinician understanding of probabilistic risk.^5^ In AD clinical trials, evidence of a statistically significant change in the Clinical Dementia Rating Sum of Boxes (CDR‐SB), an 18‐point composite measure that incorporates both cognitive and functional domains, is commonly required to support regulatory approval of therapies. The primary outcome measure is the absolute difference in change in CDR‐SB score between the treatment and placebo groups from baseline to primary outcome assessment. For example, in the CLARITY AD trial of lecanemab, the adjusted least‐squares mean change from baseline at 18 months was 1.21 in the lecanemab group and 1.66 in the placebo group, corresponding to a treatment difference of −0.45 CDR‐SB points (95% confidence interval [CI], −0.67 to −0.23; *P* < 0.001).^4^

The average between‐group difference in outcome scores provides the most direct and reliable estimate of the treatment effect for continuous outcomes in parallel‐arm trials. However, the clinical relevance of a 0.45‐point difference on the CDR‐SB scale is not readily apparent to potential prescribing clinicians, patients, caregivers, or the wider public. While this difference in CLARITY AD was statistically significant, it is not deemed to be clinically significant, that is, representing a noticeable, clinically important, and worthwhile benefit^6^ to the individual and remains a matter of debate among expert dementia clinicians and advocacy groups.

Translating this observed difference on the primary outcome measure into more easily visualized public‐facing messages is challenging.

## 3 ALTERNATIVE QUANTITATIVE INTERPRETATIONS OF THE TREATMENT EFFECT

Several alternative interpretations of the treatment effect and their intrinsic limitations have been discussed previously.^7^, ^8^ These include expressing the absolute mean difference on a clinical outcome measure as a relative percentage slowing of decline compared to placebo participants, converting continuous outcome changes into a responder proportion, or estimating the amount of time by which disease progression may have been potentially delayed.

Relative percentage slowing of decline is calculated by dividing the treatment effect by the mean decline in the placebo group. In CLARITY AD, a 0.45‐point difference relative to a 1.66‐point placebo decline was therefore reported as a 27% slowing of decline.^9^ Although this framing may superficially appear easier to communicate and understand, a relative measure can appear more impressive than the corresponding absolute differences from which it has been derived. It also carries an unevidenced implication that the treatment has potentially changed the disease trajectory beyond the measured trial period, unless the limited operating time frame and underlying calculation are clearly explained.

A related approach is expression of treatment effects as a reduced risk of progressing to a more advanced symptomatic stage or to moderate dementia. This framing may be more intuitive than a mean difference on the CDR‐SB because it maps onto clinically recognizable disease milestones. However, any such statement depends on how progression and disease stage are operationally defined, the time horizon over which risk is estimated, and whether relative estimates are accompanied by absolute event rates. Without these details, claims that treatment keeps patients in earlier symptomatic stages for longer may mistakenly be interpreted as preservation of independence or valued abilities, even when the underlying endpoint is a trial‐defined transition on a clinical rating scale.

Another means of expressing the magnitude of the treatment effect is through responder analysis, which involves classifying participants according to whether their response achieved a predefined threshold of clinically meaningful benefit.^10^ From these responder proportions, absolute and relative differences in the probability of achieving the response criterion can be calculated between treatment groups, and the number needed to treat (NNT) can be derived as the number of patients who would need to be treated for one additional patient to achieve this criterion.^11^ However, responder analyses based on dichotomizing a continuous outcome will result in loss of information and statistical power. Responder rates, as a threshold‐based transformation of continuous outcomes, are influenced by the distribution of outcomes and the selected threshold and may not accurately represent the magnitude or clinical relevance of treatment effects experienced by individual patients. In parallel‐arm trials, treatment‐specific within‐individual changes cannot be directly determined because each participant's outcome under placebo is unobserved. Furthermore, crossing a responder threshold does not necessarily indicate a meaningful benefit for an individual patient, as some patients may be classified as responders based on changes that only marginally exceed the cut‐off. As a result, responder analyses and NNT may be difficult to communicate in a way that is accurate and meaningful to the public, particularly when the underlying assumptions and thresholds used to define response are not apparent. NNT has also been shown less intuitive for patients.^5^

Further issues arise when post hoc responder analyses are applied to continuous outcomes. The application of categorical thresholds to continuous data makes the resulting estimates dependent on the choice of threshold, particularly when analyses are not prespecified. For example, a post hoc analysis of the CLARITY AD open‐label extension defined progression in disease using previously published CDR‐SB staging thresholds, with progression from mild cognitive impairment (MCI; 0.5–4) to mild AD dementia (4.5–9), or mild dementia to moderate dementia (9.5–15.1).^12^ This differed from the pre‐specified time‐to‐worsening analysis in CLARITY AD, which defined worsening as an increase of at least 0.5 points on the global CDR on two consecutive visits.^4^ Therefore, the proportion of participants who “progress” will be dependent on how the threshold is defined. These are post hoc exploratory analyses and should not be used as confirmation of efficacy; instead, they can usefully direct future research questions.

A further approach, particularly in the case of a progressively disabling condition such as AD, is the use of time‐saved estimates. These convert the absolute difference between treatment and placebo on an outcome measure at the trial endpoint into an estimated delay in disease progression, based on the speed of progression of the placebo group. This method has been argued to possess greater face validity and to communicate treatment effects more intuitively to patients and caregivers in terms of the time over which cognitive or functional abilities may be preserved.^13^ Proponents suggest that time‐based metrics may be more clinically meaningful than differences in outcome scores alone, and can also provide a means of standardizing the comparison of different clinical outcomes measures across trials.^14^

Time‐saved estimates appear attractive because they translate an abstract outcome score difference into a benefit that seems familiar and personally meaningful. However, this apparent communicative advantage is also the source of their main risk: “time saved” may be interpreted by patients and caregivers as time with preserved independence, meaningful action, or valued abilities, even when the estimate has been derived from a model‐based transformation of a rating‐scale difference and at a disease stage during which functional and cognitive differences between the beginning and end of the time‐saved period might be imperceptible. It is important to note that time‐saved analyses do not generate new information about the treatment effect, but instead translate the observed between‐group difference on a clinical outcome into an estimate on a time scale by making a number of important assumptions.

## 4 COMPLEXITIES WITH A TIME‐SAVED APPROACH

There are important statistical issues with the time‐saved approach. Backward projection to placebo (BPP) is the most commonly used method for quantifying time saved.^15^ While other approaches that rely on fewer statistical assumptions have been proposed, BPP remains the most widely used method for estimating and communicating time saved. This method transforms the primary outcome by interpreting the treatment effect along the *x* axis (time within the trial), rather than the *y* axis (clinical outcome measure).

Using BPP, the estimated mean change in the treatment arm from baseline is identified at a specified time point, such as after 18 months. A horizontal line is then projected backward from this point until it intersects the placebo trajectory, estimating the time at which the placebo group reached the same level of decline. The difference between these two time points is then calculated by subtracting the placebo time point from the treatment time point; for example, 18 – 11.4 = 6.6 months. This 6.6‐month time difference is interpreted as the estimated time saved, or as a quantification of delay in disease progression associated with treatment. This is illustrated graphically in Figure 1 using the published CDR‐SB trajectories from the CLARITY AD trial.

**FIGURE 1:**
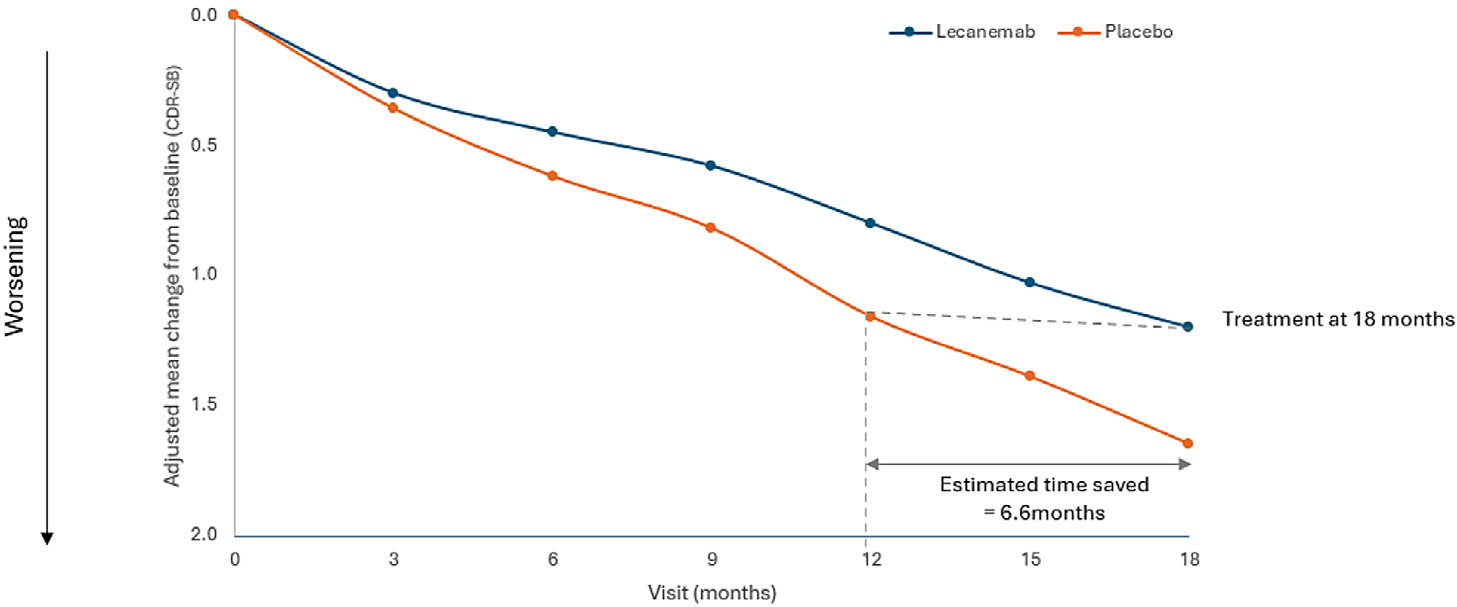
Illustration of the BPP method using digitized CDR‐SB trajectories from the CLARITY AD trial. The horizontal dashed line represents the 18‐month lecanemab outcome and is projected to the placebo trajectory. The corresponding placebo time point is then projected to the *x* axis, and the horizontal distance to 18 months represents the estimated “time saved.” The figure is intended to illustrate the BPP calculation using the published trial trajectories. BPP, backward projection to placebo; CDR‐SB, Clinical Dementia Rating Sum of Boxes.

However, BPP relies on several important statistical assumptions. First, it compares the treatment and placebo groups at different time points and therefore does not represent the observed treatment effect at the primary endpoint of the trial. In the example above, the treatment group at 18 months is compared to the placebo group at 11.4 months, rather than to the placebo group at the same 18‐month time point. Thus, BPP does not directly estimate the randomized between‐group difference at trial endpoint; it transforms the difference into an inferred difference in time.

Second, BPP disrupts the original randomized trial comparison. Randomization ensures comparability between treatment groups at the same time point in the trial; however, comparing outcomes at different time points introduces additional assumptions about the comparability of participants contributing data at those time points. Because participant attrition increases over time, the treatment group at 18 months and the placebo group at 11.4 months may differ in important ways. Such comparisons require assumptions regarding the missing‐data mechanism and that differential attrition has not introduced systematic differences between participants at those time points.

Third, BPP requires assumptions about the relationship between the treatment and placebo group trajectories. Translating the vertical difference in clinical outcome scores into a horizontal difference in time scale implies that the treatment effect can be represented as a simple shift to the left in the placebo trajectory. However, the treatment and placebo groups may follow different‐shaped trajectories and thus this assumption would not necessarily hold.

Fourth, BPP is highly dependent on the observed placebo group decline rate and trajectory of decline. By identifying the time at which the placebo group reached the same level of decline as the treatment group at 18 months and treating this difference as “time saved,” the method depends on the slope of the placebo trajectory. Estimated time saving can therefore vary substantially according to the rate and trajectory of decline in the comparator group. When the placebo group declines slowly, a small difference on the clinical outcome scale can translate into a comparatively large difference on the time scale, making a small treatment effect appear more substantial. This is particularly important when comparing treatment effects across age groups, as younger participants may decline more slowly, which can produce apparently large time‐saved estimates despite small absolute differences in clinical decline. This principle is illustrated conceptually in Figure 2, in which an identical active treatment trajectory produces different estimates of time saved when paired with different comparator trajectories.

**FIGURE 2:**
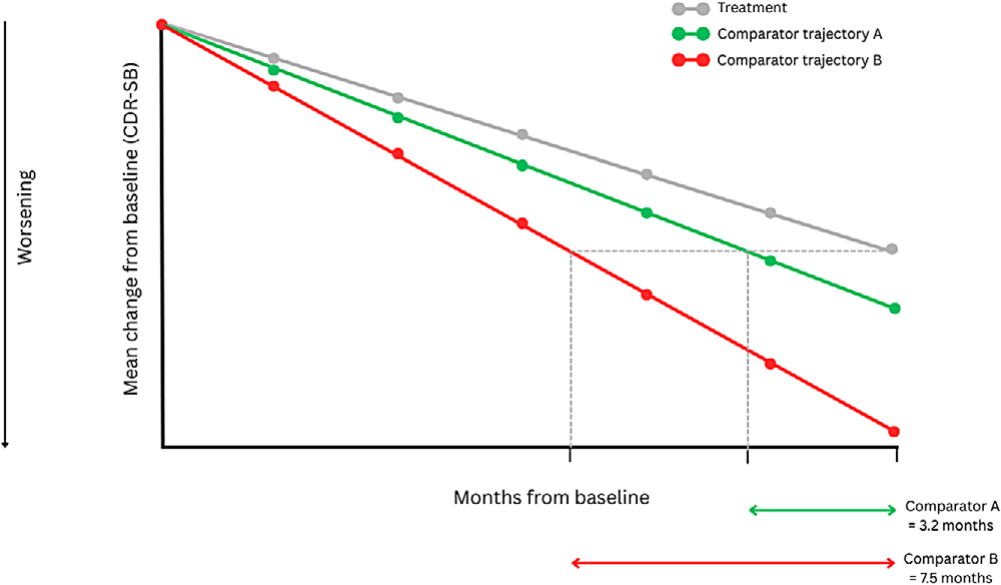
Conceptual illustration demonstrating that the estimated time saved obtained using backward projection to placebo (BPP) depends on the comparator trajectory. The treatment trajectory (gray) is identical in both scenarios, whereas the comparator trajectories differ in their rate of decline. Applying the BPP method to the same treatment trajectory yields different estimates of time saved because the comparator trajectories reach the same level of decline at different times. The trajectories are schematic and are intended solely to illustrate the mathematical properties of the method rather than observed disease progression.

Time‐saved estimates may be further vulnerable to bias when external observational cohorts are used as comparators rather than a randomized controlled placebo group. At 18 months, estimated time saved for lecanemab was reported as 6.6 months relative to placebo.^16^ In reports of the 36‐month open‐label extension, estimated time saved on the CDR‐SB was not generated by comparison to the trial placebo group. Instead, time‐saved estimates were reported as 6.8 months through BPP comparison to an Alzheimer's Disease Neuroimaging Initiative (ADNI) cohort and 8.9 months compared to a BioFINDER cohort.^16^ The difference in estimated time saved arising from use of these historic comparison groups, despite using the same treatment group, illustrates the sensitivity of time‐saved analyses to the choice of comparator population.

Time‐saved estimates may provide a useful supplementary way of summarizing trial findings, particularly when presented as model‐derived estimates. However, they should not be communicated as though they were directly observed patient‐level months of preserved function. Importantly, an estimated 6‐month delay does not mean that treated participants experience a level of function equivalent to accumulated benefit during the whole trial period (this would only be true at the 18‐month point) or that an additional 6 months before progressing will be experienced. Communicating time savings in this way is extremely misleading and exaggerates potential benefits egregiously. Demonstrating such a delay would require a pre‐specified and defined time‐to‐event analysis, which has not been available for any of the licensed drugs.

## 5 TIME SAVINGS: 6 MONTHS OF WHAT?

Even if time‐saved estimates are considered a valid statistical approach to reporting trial results, an important question remains: What does an apparent 6‐month delay in disease progression in a trial population of individuals with MCI and mild dementia actually represent?

A 6‐month time saving does not imply a 6‐month period free from symptoms or a 6‐month pause in disease progression. AD progresses gradually over many years, and progression of symptoms at the MCI and mild dementia stages is relatively slow. Longitudinal studies suggest that changes in cognitive measures over a 6‐month period are small, with an amyloid‐positive cohort of individuals with diagnoses of MCI or mild dementia showing average Mini‐Mental State Examination (MMSE) changes of 0.6 MMSE points over 18 months.^17^ Consequently, a 6‐month time saving, transformed from the small drug–placebo differences observed on the CDR‐SB with lecanemab, corresponds to a very small difference in cognitive and functional status between time points, for example, from a mild degree of cognitive and functional impairment to an only slightly more impaired state that may be clinically hard to perceive.

The meaning of “time” is therefore critical. A time‐saved estimate may refer to time on a rating scale, namely a model‐derived delay in reaching a particular CDR‐SB score. It may be interpreted more broadly as time in an earlier disease stage, although this may not be directly measured or convincingly statistically confirmed. Patients and caregivers, however, may understandably and potentially incorrectly interpret it as time with preserved valued abilities: driving, managing finances, living independently, participating in family life, or recognizing loved ones. These are the outcomes that matter most to patients and families, but they are also the outcomes least directly demonstrated by model‐derived estimates of time saved in patients with MCI or mild AD dementia.

It could be argued that a disease‐modifying treatment potentially shifts the experience of disability caused by dementia backward, so that time saved at the MCI or mild dementia stage is effectively transferred to later stages, when loss of independence and ability to perform highly valued activities would otherwise have occurred. Interpretation of clinical significance of time‐saved estimates is thus highly dependent on whether treatment effects are symptomatic, short‐acting, or sustained disease‐modifying effects. For a purely symptomatic therapy, treatment and placebo trajectories would be expected to converge over time, with patients ultimately reaching similar levels of cognitive and functional impairment, so that apparent time‐savings benefits would be lost by the time ability to perform highly valued activities is threatened. By contrast, a disease‐modifying therapy would be expected to produce sustained separation between trajectories, with preservation of time savings sufficient to make important differences to the trajectory of loss of function and independence.

Demonstrating true clinical disease‐course modification requires evidence that treatment produces a sustained alteration in the underlying disease course. One of the main challenges to this is distinguishing disease modification from symptomatic benefit.^18^ A treatment that leads to symptomatic improvement or temporary slowing of symptom progression can make treated patients appear to be at an earlier stage of disease. This is reflected in clinical trials of cholinesterase inhibitors, which produced symptomatic effects in cognition and function, yet do not alter the underlying disease cause.^19^, ^20^ Delayed‐start trial designs have traditionally been proposed as one important means of supporting such claims of disease modification. However, randomized delayed‐start data are currently lacking for amyloid‐lowering therapies.^21^, ^22^ Amyloid lowering provides evidence of good biological target engagement, and clinical trajectories separated over the randomized trial period. However, whether model‐derived time savings are preserved into later, patient‐salient outcomes would require evidence that cannot be derived from the primary randomized trial period.

Consequently, it remains unclear whether reported time savings do translate into a meaningful delay in symptoms at a future point. It would be unfair to offer such a promise to patients and caregivers when they are making decisions about treatment.

## 6 HOW ARE TREATMENT BENEFITS COMMUNICATED QUALITATIVELY?

Quantitative trial results are often translated into qualitative descriptions to improve accessibility. These benefits are communicated through a range of channels, including statements made by charities, pharmaceutical companies, traditional media, and social media. Similar qualitative descriptions are often used, including descriptions of individuals who have maintained independence, attributed to treatment, with the promise of more time spent in an earlier stage of disease and consequent preservation of valued aspects of quality of life.

No single communication channel determines public understanding. Rather, expectations are shaped cumulatively through press releases, advocacy materials, professional commentary, media reporting, social media, and patient stories. When these channels converge on simplified claims of preserved independence or extra time without disability, the distinction between observed trial effects and inferred patient‐level benefit can become potentially misleading. The consistency and amplification of this messaging increase salience and credibility among clinical and lay audiences.

For example, treatment benefits have been described as “extending independent living” or providing patients with “more time to participate in daily life and live independently.”^23^, ^24^ These descriptions are tangible and attractive for a lay audience but may not fairly reflect the conclusions that can be drawn from the outcomes measured in the trials. They risk translating a model‐derived or scale‐based delay into a claim about independence, even when effects on independence, driving, institutionalization, or maintenance of specific valued activities were not directly demonstrated as delayed by the trial evidence.

Another communication strategy that can be powerful and highly relatable is the use of individual patient and caregiver narratives. For example, the Leqembi website carries a dedicated “Patient Stories” section, where members of the public can watch video testimonials from individuals with AD who have received treatment and their family members.^25^ These narratives may help provide a lived‐experience view of treatment effects. However, such anecdotal accounts are not representative of average objective treatment effects in clinical trials, and their selection and use risks overemphasizing the clinical benefits of treatment if not accompanied by balanced information about uncertainty and variability of individual outcomes.

Qualitative research can provide additional insights into the importance patients and caregivers place on exactly how treatment benefits are communicated. In semi‐structured interviews with individuals attending memory clinics with biomarker or imaging evidence of AD who were asked about their views on lecanemab, participants placed considerable trust in information obtained from advocacy organizations and internet sources.^26^ Decisions regarding treatment initiation were largely driven by hopes for benefit, expectations regarding treatment effectiveness, and the perceived existential threat posed by severe dementia. This indicates the understandable vulnerability of people with dementia to the effects of any exaggeration of the potential benefits of treatment, and the importance of precision and accuracy in conveying interpretations of clinical trial results or explanations of how those benefits might reasonably translate to the experience of dementia for individuals.

Communication may also frame treatment as a means of restoring agency, allowing patients to “take action,” “alter their future,” or “invest” in a combined therapeutic and lifestyle program. Such language may be empowering and consistent with shared decision making when balanced by realistic information. However, it may also amplify expectations if the possibility of action is not clearly distinguished from the magnitude, uncertainty, burden, and limits of the treatment effect demonstrated in trials. Further examples of potential claims, explanations, and their underlying evidence base are provided in Table 1.

**TABLE 1:** Examples of communication statements and their evidential basis in Alzheimer's disease clinical trials.

Evidential category	Claim	Communication risk	Preferable wording
Directly supported	*Treated participants worsened less, on average, than placebo*.	May be interpreted as predicting individual benefit.	*People receiving treatment continued to worsen, but on average worsened more slowly than those receiving placebo*.
Requires qualification	*Treatment slowed decline by a relative percentage*.	May exaggerate perceived benefit.	*The relative percentage should be presented with the absolute difference on the trial scale, the denominator, and the trial duration*.
Reasonable interpretation	*Treatment may keep selected patients at an earlier stage for longer*.	May imply preserved independence.	*Treatment may reduce progression to a more advanced stage during the trial, but this does not necessarily mean preserved daily abilities*.
Model‐derived estimate	*Treatment delayed progression by X months*.	May be mistaken for observed decline‐free time.	*This is a model‐based estimate and does not imply X months without decline*.
Not demonstrated	*Treatment provides extra months of independence or valued abilities*.	Converts trial‐scale differences into unproven patient‐level promises.	*This should not be claimed unless independence or specific valued abilities were directly measured and shown to be preserved*.
Incorrect	*The disease is paused or put on hold*.	Scientifically incorrect and misleading.	*The disease was not paused; treated participants still declined, but more slowly on average*.

## 7 Conclusion

There is a range of approaches to communicating AD clinical trial results and how these should shape the expectations of patients and caregivers, encompassing both quantitative and qualitative methods. Primary trial outcomes are frequently translated into alternative quantitative interpretations of the treatment effect, such as relative slowing of decline and time‐saved approaches. These approaches may improve accessibility and may help non‐specialist audiences understand why statistically significant differences on clinical rating scales matter.

However, communication must remain precise and should not extend beyond the evidential bounds of what the trials were designed to assess and have demonstrated. Communication should distinguish clearly between modification of amyloid pathology and modification of the clinical disease course. Amyloid‐lowering therapies demonstrably reduce brain amyloid, but whether this translates into sustained clinical disease modification remains uncertain.

Public communication should begin with the observed absolute between‐group effect in the randomized trial, specify the population and time horizon, and then clearly identify any further statement as a relative transformation, threshold‐based responder analysis, model‐derived time‐saved estimate, or patient‐salient extrapolation. Claims that treatment provides additional months of independence, preserves specific valued abilities, or puts disease progression “on hold” should not be made unless those outcomes were directly demonstrated. In a field shaped by fear, hope, and urgent clinical need, this evidential hierarchy is essential for informed consent and public trust.

## AUTHOR CONTRIBUTIONS

Jemma Hazan, Simone Salemme, Kathy Y. Liu, and Robert Howard conceptualized the manuscript. Jemma Hazan wrote the original draft. Simone Salemme, Jemma Hazan, Kathy Y. Liu, and Robert Howard reviewed and edited the manuscript. All authors reviewed and approved the final manuscript and visualizations.

## CONFLICT OF INTEREST STATEMENT

J.H. reports no disclosures relevant to the manuscript. S.S. reports consultancy for Italian National Institute of Health and the World Health Organization, as well as travel and attendance scholarships from the European Academy of Neurology, Alzheimer's Disease International, and the Italian Society of Neurology. K.L. reports no disclosures relevant to the manuscript. R.H. reports unpaid advisory board work for Synaptogenix in the past 5 years. The authors declare that none of these interests is relevant to the content of this Perspective article. Author disclosures are available in the Supporting Information.

## Supporting information



Supporting Information
